# Lessons Learned from Implementing a Wet Laboratory Molecular Training Workshop for Beach Water Quality Monitoring

**DOI:** 10.1371/journal.pone.0121214

**Published:** 2015-03-30

**Authors:** Marc Paul Verhougstraete, Sydney Brothers, Wayne Litaker, A. Denene Blackwood, Rachel Noble

**Affiliations:** 1 The University of Arizona, Mel and Enid Zuckerman College of Public Health, Tucson, Arizona, United States of America; 2 University of North Carolina-Wilmington, Wilmington, North Carolina, United States of America; 3 National Oceanic and Atmospheric Administration, National Ocean Service, Center for Coastal Fisheries and Habitat Research, Beaufort, North Carolina United States of America; 4 University of North Carolina-Chapel Hill, Institute of Marine Science, Morehead City, North Carolina, United States of America; University of Western Sydney, AUSTRALIA

## Abstract

Rapid molecular testing methods are poised to replace many of the conventional, culture-based tests currently used in fields such as water quality and food science. Rapid qPCR methods have the benefit of being faster than conventional methods and provide a means to more accurately protect public health. However, many scientists and technicians in water and food quality microbiology laboratories have limited experience using these molecular tests. To ensure that practitioners can use and implement qPCR techniques successfully, we developed a week long workshop to provide hands-on training and exposure to rapid molecular methods for water quality management. This workshop trained academic professors, government employees, private industry representatives, and graduate students in rapid qPCR methods for monitoring recreational water quality. Attendees were immersed in these new methods with hands-on laboratory sessions, lectures, and one-on-one training. Upon completion, the attendees gained sufficient knowledge and practice to teach and share these new molecular techniques with colleagues at their respective laboratories. Key findings from this workshop demonstrated: 1) participants with no prior experience could be effectively trained to conduct highly repeatable qPCR analysis in one week; 2) participants with different desirable outcomes required exposure to a range of different platforms and sample processing approaches; and 3) the collaborative interaction amongst newly trained practitioners, workshop leaders, and members of the water quality community helped foster a cohesive cohort of individuals which can advocate powerful cohort for proper implementation of molecular methods.

## Introduction

Monitoring of recreational waters for human pathogens around the world largely depends on the cultivation of fecal indicator bacteria [1–4]. This process requires an incubation period of 18–48 hours [5,6]. This long incubation period can result in prolonged exposure to pathogens prior to obtaining the information required to issue a closure notice [7].

The United States Environmental Protection Agency (USEPA) recently recommended the use of a rapid molecular method for monitoring fecal indicator bacteria at beaches throughout the nation [1]. This method measures *Enterococcus* spp. using quantitative Polymerase Chain Reaction (qPCR) [8]. It was previously demonstrated that using rapid, qPCR-based methods, a statistically significant relationship exists between *Enterococcus* spp. density in marine and fresh waters impacted by human feces and gastrointestinal illness [9–12]. The USEPA method 1611 uses qPCR to detect *Enterococcus* spp. deoxyribonucleic acid (DNA) extracted from water samples and produces water quality results in 2–4 hours [13]. Other methods for quantification of *Enterococcus* spp. in water have been developed and show similar statistically significant relationships to beach water quality [12,14].

Significant constraints exist for laboratories attempting to implement rapid molecular techniques for beach monitoring [15]. The challenges exist largely because water quality microbiology laboratories use classical microbiology techniques such as membrane filtration, multiple tube fermentation, and defined substrate technologies to quantify fecal indicator bacteria. These conventional methods require lengthy incubation. They also rely on simple filtration or basic processing of samples that includes working with larger volumes of sample water (>10 ml). Rapid molecular methods use similar equipment for the initial sample processing, but the majority of molecular methods require precise techniques and complex equipment. Specific challenges to the implementation of a successful rapid qPCR method include proficiency and repeatability of small-volume pipetting, prevention of cross-contamination, and effective enumeration of gene copy based qPCR standards. Furthermore, major interpretive differences for beach management actions exist in qPCR derived data [13]. The purpose of this manuscript is to discuss the challenges encountered and lessons learned from implementing the first recreational water quality-focused, rapid methods workshop for training professionals in the community.

## Workshop Approach

The workshop leaders and authors of this manuscript identified the program evaluation presented in this manuscript did not meet the established criteria requiring ethics committee approval [16–18]. Discussion with a University Human Subjects Protection Program confirmed the manuscript is not human research but rather program evaluation and thus, IRB approval was not sought. Although IRB approval was not required, participation protection measures were applied during the workshop and manuscript development. Participants sought out, enrolled, paid registration fees, and participated in the workshop on their own accord. Participant consent to participate in this hands-on training workshop was implied during the written registration process. Explicit written consent was not sought from participants to use workshop results as the data collected was for program evaluation purposes only, not human research. Participant registration, workshop lists, workshop results, and workshop evaluations have not been publicly available including to the workshop website or original funding agency website. This manuscript utilized pooled results from efforts performed both individually and in groups. After pooling all de-identified data, analyses were performed as a workshop whole.

A “Molecular Training Facility” (MTF) was developed at the University of North Carolina’s Institute of Marine Sciences (UNC-IMS). The mission of the MTF is to train water quality and food science professionals in new molecular methods for improved monitoring and quantification of microbiological targets. The facility organization and workshops were conducted by leaders in the field of rapid molecular methods for water quality, food science, and aquaculture. All of the leaders have had previous experience training users in water quality science.

The first training workshop was devoted to the use of rapid qPCR for monitoring recreational water quality. It was held at UNC-IMS from March 10–15, 2013. The workshop included an optional introduction to PCR, qPCR, and pipetting proficiency attended by nine of the 18 workshop participants. The affiliation of participants included academic professors [2], government employees [11], private industry representatives [2], and graduate students [3]. Participants from all regions of the USA were represented, many traveling greater than 1,000 miles to attend the workshop. Upon completion of this workshop, participants were expected to: 1) understand qPCR theory and practices; 2) implement rapid methods approved by the USEPA to monitor recreational waters, and 3) perform necessary data calculations to generate and interpret meaningful results.

In order to focus the workshop at a level suitable for all participants, a pre-workshop skills assessment survey, completed by all participants prior to arrival, was included with the two-page registration form. This survey aimed to determine the level of participants’ laboratory skills including pipetting, aseptic techniques, and microbial and molecular biology experience. The eight-question survey included five multiple-choice questions and three short answer questions The skills evaluation was used to create collaborative groups, pairing more-advanced with less-advanced participants. This cross-level approach was taken to help impart participant experiences and reduce the power imbalance associated with a strictly leader-participant teaching approach [19,20].

Prior to arriving at the workshop, participants were mailed background reading materials and exercises to practice their molecular and laboratory calculations. The practice problems consisted of simple unit conversions, general equations, and molecular theories aimed at introducing participants to laboratory concepts and theories that would be further explained during the workshop.

Upon arrival, each participant was provided a workshop manual that contained participants’ contact information, a workshop schedule (overview and detailed), and a detailed training guide. The training guide included important definitions and acronyms, laboratory techniques (i.e., safety awareness and rules, aseptic techniques, and pipetting proficiency standards), Minimum Information for Publication of Quantitative Real-Time PCR Experiments (MIQE) guidelines, a microbial source tracking summary, detailed laboratory qPCR techniques for *E*. *coli* and enterococci, platform operation instructions, DNA extraction procedures, and references/resources.

The workshop focused on training participants to use three different assays. These assays are currently used, or have the potential to be used, for recreational water quality monitoring throughout the United States. Training included two assays specific for quantification of *Enterococcus* spp. (USEPA Method 1611 and Scorpion *Enterococcus* spp. SampleReady Assay) and one assay specifically designed for quantification of *E*. *coli* (Scorpion *E*. *coli* SampleReady Assay). The recently updated USEPA recreational water quality criteria recommended the use of Method 1611 [1,8]. The *Enterococcus* spp. SampleReady Assay was previously tested and published [12,14]. The *E*. *coli* Scorpion SampleReady Assay is currently approved for monitoring freshwater beaches in Wisconsin, Ohio, and Michigan (e.g. [21]).

Three qPCR platforms were employed during the workshop to run the three selected assays. All three assays can be conducted successfully on each of the three platforms, but for workshop simplicity, the Applied Biosystems StepOne Plus Real-Time PCR system was used to conduct USEPA Method 1611 and the Bio-Rad CFX96 Touch and Cepheid SmarCycler II systems were used to demonstrate the Scorpion SampleReady assays. All participants produced their own cell standards, conducted a standard curve on each qPCR platform (n = 3), and used the data generated to determine the concentration of *Enterococcus* spp. in the original sample. Additionally, each participant analyzed their own data using calculation templates and learned to troubleshoot qPCR problems.

Each participant was provided with a workshop kit which contained all the required laboratory materials for the day. The kits were inspected and given to the participants at the start of the workshop. Each student was then required to use a check list to identify all the items in the kit as a way to become familiar with the required equipment. At the conclusion of the workshop, all kits were rechecked for completeness and to estimate total material usage. Prior to the start of each activity, leaders and assistants added material (e.g. aliquots of molecular grade water, reagents, and primers/probes) to the kits depending on the activity requirements. The purpose of these kits was not to eliminate the important skills of setting up a workstation and proper labeling techniques, but to expedite the laboratory training, ensure sufficient time for learning the molecular techniques, and reduce contamination of stock solutions from multiple participant entries. Participants were responsible for setting up their bench space and cleaning before and after all exercises. To reduce the possibility of cross contamination, leaders cleaned all equipment (e.g. microcentrifuge tube racks, bench tops, and micropipettes) at the start of each day.

Lectures covered key concepts and included: PCR and qPCR theory; Quality Assurance/Quality Control; qPCR quantification and standards; data analysis and data interpretation; troubleshooting qPCR results; inhibition identification in samples; MIQE guidelines [22]; and microbial source tracking applications.

Upon completion of the workshop, participants were provided an evaluation questionnaire. The anonymous questionnaire surveyed the expectations of the participants prior to the workshop and evaluated the knowledge gained during the workshop, overall workshop experience, workshop material, workshop environment and organization. The evaluation was comprised of several Likert-type scaled questions and had five short answer/open ended questions aimed at supporting the Likert-type scaled questions. Evaluations also included eight questions for each leader to help leaders improve upon their strengths and weaknesses. Responses for Likert-type scaled questions were evaluated using percent agreement. Analysis of short answer/open ended questions was accomplished by first creating categories of the various response themes and then assigning at least one category to each response. The evaluation was aimed to get participant feedback on the following aspects: 1) overall workshop experience; 2) workshop environment; and 3) workshop material. Table 1 summarizes the questions (Q1-Q21) asked which address these aspects and their respective response type (i.e., Likert-type scale or short answer). The evaluation was created with the assistance of survey experts from the University of North Carolina and NOAA and will be made available for similar evaluation purposes upon request.

**Table 1:** Summary of questions on the written workshop evaluation.

Evaluation topic	Question number	Question	Response type
Overall workshop experience	Q1	Overall, how satisfied were you with the speakers/presenters?	Likert-type scale
	Q2	Overall, how satisfied were you with the workshop facilities?	Likert-type scale
	Q3	Overall, how satisfied were you with the laboratory information presented?	Likert-type scale
	Q4	Overall, how satisfied were you with the workshop organization?	Likert-type scale
Workshop environment	Q5	The workshop was well organized.	Likert-type scale
	Q6	The atmosphere of the workshop was professional.	Likert-type scale
	Q7	The lodging arrangements were clean and appropriate for this venue.	Likert-type scale
	Q8	The food selection was appropriate, on time, and enjoyable.	Likert-type scale
	Q9	Transportation during the workshop was on time, comfortable, and drivers were courteous.	Likert-type scale
Workshop material	Q10	The workshop increased my knowledge of molecular techniques.	Likert-type scale
	Q11	The workshop was well paced within the allotted amount of time.	Likert-type scale
	Q12	The workshop goals were clearly stated.	Likert-type scale
	Q13	The workshop protocols were clear and useful.	Likert-type scale
	Q14	Questions and concerns were addressed appropriately.	Likert-type scale
	Q15	The presenters provided for a variety of learning styles.	Likert-type scale
	Q16	I am comfortable teaching the material presented in this workshop.	Likert-type scale
Overall workshop experience;	Q17	What were your expectations before the workshop? Were they met?	Short answer
Workshop environment;	Q18	What would you like to see at a future workshop?	Short answer
Workshop material	Q19	What did you like most about the workshop?	Short answer
	Q20	What did you like least about the workshop?	Short answer
	Q21	In what ways could this workshop be improved?	Short answer

## Lessons Learned

**Fig 1:**
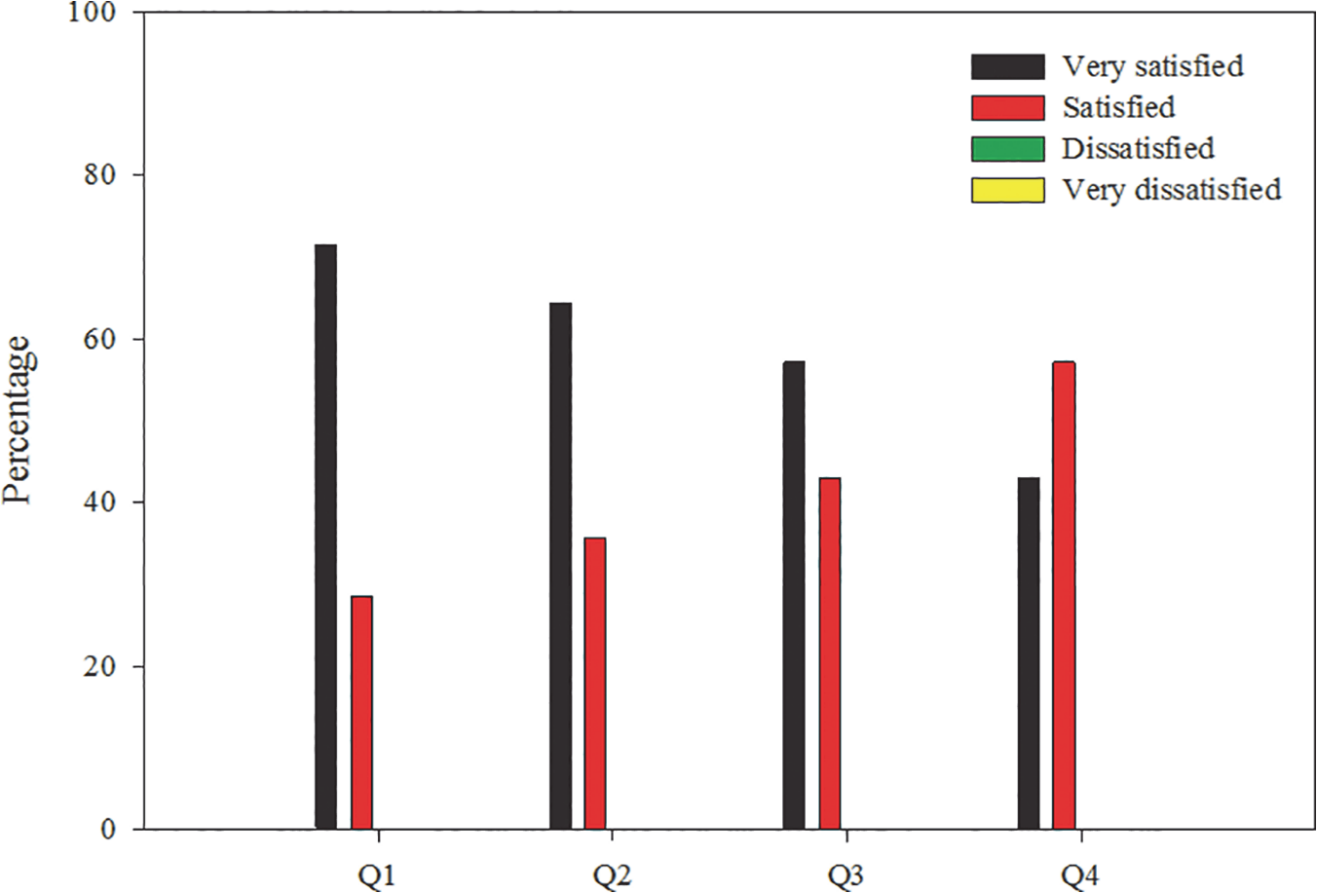
Participant written evaluation responses to questions focused on the overall workshop experience. Q1: Overall, how satisfied were you with the speakers/presenters?; Q2: Overall, how satisfied were you with the workshop facilities?; Q3: Overall, how satisfied were you with the laboratory information presented?; Q4: Overall, how satisfied were you with the workshop organization?

The feedback received from the evaluation questionnaire was constructive and generally positive, as depicted in Fig 1. The following discussion incorporates feedback from participants (personal communication and questionnaire responses) and leaders (personal communication).

### Workshop Environment

**Fig 2:**
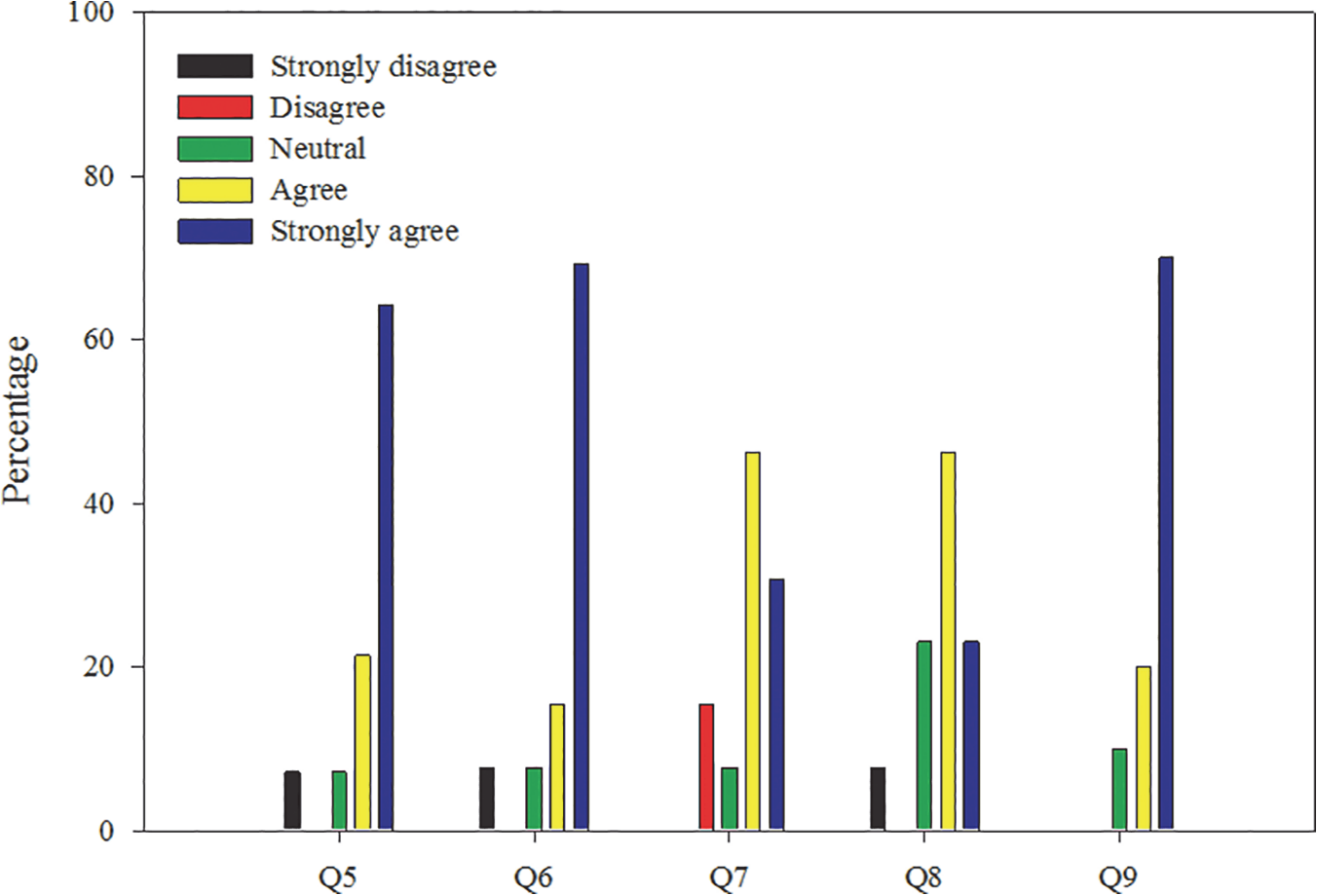
Participant written evaluation responses focused on the workshop environment. Q5: The workshop was well organized; Q6: The atmosphere of the workshop was professional; Q7: The lodging arrangements were clean and appropriate for this venue; Q8: The food selection was appropriate, on time, and enjoyable; Q9: Transportation during the workshop was on time, comfortable, and drivers were courteous.

The workshop was held at UNC-IMS (Morehead City, NC, USA), an off-campus research laboratory and teaching facility located on Atlantic Ocean coastline. UNC-IMS facilities include approximately 60,000 square feet of research space, a running sea water system that feeds indoor and outdoor experimental facilities, outside experimental ponds, meeting rooms, and a maintenance and fabrication facility. The workshop environment and accommodations received mixed reviews from workshop participants (Fig 2). Many participants (66%) felt that the laboratory space was inadequate for the number of participants (Q2, Q18, Q20, and Q21). Written feedback indicated that participants would have benefited from more than approximately 8 square feet of bench space and room for each person to sit down during laboratory activities. Therefore, we suggest future workshops cap participation at a size proportional to the smallest room used during the workshop. We also suggest arranging laboratory, lecture, and break rooms in close proximity in order to reduce idle time between activities.

### Workshop preparations

**Fig 3:**
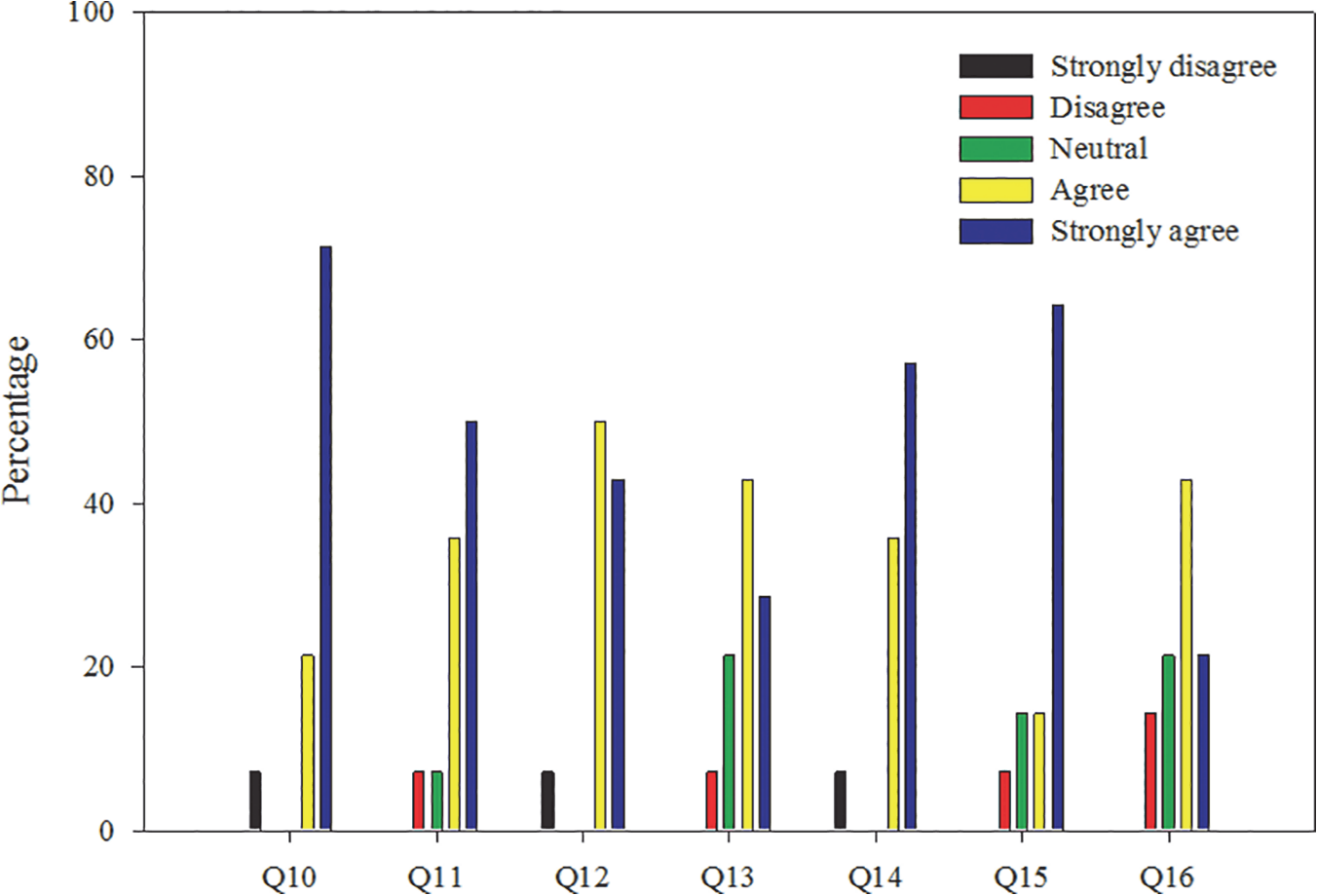
Participant written evaluation responses focused on the workshop material. Q10: The workshop increased my knowledge of molecular techniques; Q11: The workshop was well paced within the allotted amount of time; Q12: The workshop goals were clearly stated; Q13: The workshop protocols were clear and useful; Q14: Questions and concerns were addressed appropriately; Q15: The presenters provided for a variety of learning styles; Q16: I am comfortable teaching the material presented in this workshop.

The most important aspect to ensure a successful workshop was preparation. These preparations resulted in an overall positive experience (Fig 3). Finalization of the schedule defined the workshop and allowed for efficient laboratory and lecture preparations. Since this was the first workshop in an ongoing series, it was difficult to estimate the amount of time each participant would require to complete any given activity. For this reason, additional time was built into the workshop schedule and laboratory activities were buffered by lectures and breaks. When appropriate, timers were used to keep participants on task and on time. Scheduled breaks were used to prepare and clean between key laboratory steps, limiting cross contamination of equipment and reagents with genomic DNA. This practice resulted in minimal contamination during the workshop, as indicated by 87% of negative control curves exhibiting no amplification.

Three weeks prior to the start of the workshop, two volunteers completely new to molecular methods helped test the timing and laboratory aspects of the workshop. The benefit of this practice was to highlight activity bottlenecks and validate methods, reagents, and platforms. Identified bottlenecks were addressed when possible, but in the instances where they could not be avoided (i.e., limited equipment or space), participants were paired, based on the pre-workshop skills survey. Additional qPCR platforms were needed to eliminate bottlenecking during the hands-on portion of the workshop. Arrangements were made with each vendor to have equipment delivered and setup at least one week in advance to perform Quality Assurance/Quality Control. Quality Assurance/Quality Control should be performed on all reagents (e.g.,. primers, probes, cell standards, samples) at least two months before the workshop. While this deadline was missed for the first workshop, such a timeframe would allow sufficient time to reorder, validate, aliquot, and label new products.

Lectures covered multiple topics, including: a summary of recreational water quality criteria by USEPA staff; overview of PCR and qPCR; inhibition and interference; qPCR QA/QC techniques; MIQE guidelines; and Microbial Source Tracking. Presentations were prepared and pre-screened by the leaders two weeks before the workshop. All presentations, except the USEPA presentation, used the same format and hardcopies were included in participant’s binders. Lectures of 15–30 minutes were generally concluded with 15–30 minutes of question and discussion. The 60 minute USEPA session was conducted via distance learning using a teleconferencing system and was not tailored to the workshop or participants. Three participants indicated the USEPA presentation was the least liked aspect of the workshop (Q20); indicating the need for modifying presentations to specific audiences and learning styles. We suggest presenting material in multiple forms to reach the largest number of learning styles and improve learning efficiency and effectiveness [23]. Consistent formatting and clear labeling on all files, folders, handouts, and protocols was used for easier participant interpretation. Presentations, methods, and protocols used during the workshop were provided to participants in electronic and hard copies to distribute amongst their home laboratories.

Laboratory kits were prepared prior to the workshop for each participant or group. While the leaders acknowledge that laboratory setup and labeling are critical skills that need to be taught, the efforts were beyond the scope of time allotted during this workshop. The laboratory kits contributed to a smooth operation at the beginning of each day, reduced contamination of stock solutions, and allowed for the maximum amount of hands-on laboratory time dedicated to learning molecular methods. Workshop efficiency would have improved if everything was pre-labeled including pipette tip boxes and microcentrifuge tubes. An applied rule of thumb was to allocate 1.5 times the amount of required disposable materials and reagents to compensate for spills and contamination.

### Workshop Materials

Throughout the workshop, it became apparent that participants greatly benefited from enhanced workshop materials such as laboratory diagrams and truncated methods. Conceptual maps, abbreviated methods, and laboratory diagrams were developed after the workshop following guidelines from [24]. Additionally, a daily walkthrough of laboratory protocols and displayed flowcharts of key points would have improved comprehension of applied methods. We suggest a daily summary of the schedule and activities first with the leaders prior to participant arrival and then again with the participants at the beginning of each day. Furthermore, current microbial and molecular biology protocols, appearing in various forms, are too cumbersome and detailed for rapid dissemination. We believe these protocols (e.g. USEPA Method 1611 and Standard Methods) require simplification as streamlined protocols increase accuracy and decrease analytical variability and method-related errors [14].

As in many fields, several words and acronyms are used interchangeably and participant feedback indicated confusion with the vocabulary (Q20). Despite our best efforts to provide a comprehensive list of definitions and acronyms prior to the workshop, these obstacles remained but could be overcome through continuous literature exposure, hands-on experiences, and writing, evaluating, and discussion of the concepts [25–27]. This issue is prevalent in the water quality science community and has been highlighted in previous publications [28,29]. To create consistent use of terms and acronyms, continual and cross-field efforts are required.

Commercial industry vendors (Bio-Rad, BioGx, and Life Technologies) were involved with the workshop. The representatives of these companies led lectures and platform demonstrations prior to the use of their respective platform in the laboratory, provided hands-on demonstrations, donated materials for use during the workshop, and participated in a vendor show at the end of the workshop. While personal communication with participants indicated a positive review of industry involvement, future written evaluations should incorporate participant, vendor, and instructor assessment of industry involvement. This feedback could identify if the industry interactions improved the educational and organizational outcomes of the workshop.

### Hands-on Laboratory Training

Extensive workshop time was devoted to the hands-on laboratory training with less time focused on lecture and classroom exercises. Written evaluation responses to the most enjoyed aspects of the workshop (Q19) included 11 of 14 participants (79%) mentioning laboratory “hands-on” time/training. During the final laboratory exercise, participants worked with real-world samples and processed them through calculation of results and data interpretation. On the written evaluation responses (Q21), three respondents felt they would have benefited further from additional training focused on beginning to end sample processing. Participant feedback also indicated interest in longer workshop days to provide additional hands-on laboratory training and real world sample data interpretation (Q21). We suggest future workshops incorporate multiple activities that combine all workshop concepts into a continuous exercise (i.e., real world simulation).

Environmental world samples were collected from three locations: an oligohaline portion of the Neuse River Estuary; 32^nd^ Street stormwater outfall in Morehead City, NC; and the stormwater retention pond at UNC-IMS. The samples were collected in order to represent a range of salinities, turbidities, and qPCR inhibitory compounds.

Participants demonstrated apprehension about bench space setup including placement of kits, equipment (vortex, centrifuge, etc.), and samples (personal communications and leader observations); likely as a result of the unfamiliar environments. It was proposed that a bench space diagram or overhead bench shelves be utilized to reduce confusion.

### Data entry and calculations

Training success depended on each participant understanding routinely used laboratory equations and methods for preparation of reagents and materials. However, few participants completed the pre-workshop assigned reading and problem sets, requiring additional classroom time for explanation. Future workshops should assume that all participants have no background knowledge of the materials, basic equations, or approaches. The flipped classroom approach assumes that participants have read for understanding and completed all pre-workshop assignments prior to entering the classroom. The alternative assumption should be made that participants did not complete any of the assignments and all components critical to the workshop success should be thoroughly covered at the beginning and reviewed throughout the workshop. Errors were identified in the data calculation spreadsheets and some protocols were not wholly concise and clear. These oversights were emphasized in participant’s written evaluation responses supporting their Likert-type scaled responses to Q1 (n = 2), Q3 (n = 2), Q13 (four participants did not agree that protocols were clear and useful), Q18 (n = 1), Q20 (n = 3), and Q21 (n = 2); highlighting the need for extensive testing of all workshop material. We suggest allocating ample time to cover critical workshop concepts on the first day and follow up with critical thinking questions to demonstrate comprehension. While this may seem to be a basic finding, it is vital to the proficiency of the qPCR user.

### Rapid Methods Practitioner Cohort Development

The fundamental aim of this workshop was to promote interactions not only among leaders and participants, but also among participants. Horizontal peer support [30], or participants supporting one another, was deemed essential to maximize the potential of training. Peer training support is effective as it enables participants to engage more freely and fully [20] and reduces the power imbalance from leader-participant interactions. Peer teaching, knowledge sharing, and development are vital to many scientific laboratories but are often limited by financial resources of training facilities and personnel of participant laboratories. In the workshop, it became apparent that there was a lack of adequate time dedicated to fostering peer learning and relationships in the workshop; similar to the limitations of peer learning during training exercises identified elsewhere [31–33]. These limitations can be overcome by providing additional laboratory time during the workshop with limited direct leader oversight. Although this would be difficult to support from a financial and personnel standpoint, the participants would also benefit from post-workshop support in their respective laboratories.

We recognize that the participants’ understanding of qPCR concepts improved as the workshop progressed, as identified through personal feedback and the evaluation questionnaire. By the end of the workshop, the participants became more confident in their abilities to return to their respective organizations and become the trainers. Nearly all survey respondents (93%) reported an increased knowledge of molecular techniques (Q10) and 64% said that upon leaving the workshop they would be comfortable teaching the material presented to others (Q16). Additionally, participants felt that the training and knowledge gained at the workshop allowed them to expand their newly developed skills beyond recreational water quality monitoring to include more advanced methods (Q17 and personal communication) which employ the same concepts, such as molecular source tracking. However, the perceived increased knowledge of qPCR concepts did not directly translate into an overall improvement of qPCR efficiency (Table 2). This suggests that parts of the workshop need to be reconsidered as the primary goal was to improve performance. Increased perceived knowledge without demonstrated improved technical accuracy, as shown via the standard curves, highlights improved declarative understanding only.

**Table 2:** Summary of standard curve qPCR results as a progression during the workshop.

Run	Number of groups/participants	Assay	R^2^	Efficiency
1	12	*Enterococcus*	0.91	104.8
2	15	*Enterococcus* FAM	0.93	113.9
		*Enterococcus* Scorpion	0.89	92.72
3	4	*Enterococcus* Scorpion	0.99	84.69

## Future Workshops

The demonstration workshop, evaluation questionnaire, and personal feedback from participants and leaders taught us that the biggest impediments to the universal adoption of qPCR will be variability across users and implementation cost. While there is no immediate solution for this, the technology is improving and qPCR techniques are becoming cheaper and more user-friendly.

We suggest future workshops include: Reduced lecture time

1. Increased hands-on laboratory time.
2. Processing of environmental samples from collection through data/result interpretation.
3. Implementation of a vendor show scheduled during the middle of the workshop to provide participants with unrestricted time to discuss equipment capabilities and cost
4. Enrollment sized appropriately based on the smallest workshop room so that each participant has adequate bench space.
5. Increased critical thinking assignments.
6. Inclusion of peer relationship development activities.

## Key Suggestions

An evening group discussion was held once during the workshop. Based on personal communications with workshop participants, this discussion was well received and participants claimed it was extremely helpful. This discussion allowed participants with different requirements to ask questions regarding methods and equipment. During future workshops, we suggest a daily summary and group discussion to reiterate important concepts applied that day and to elicit further inquiries. The discussion need not be long (e.g., 30 minutes) or mandatory, but such a gathering offers participants the option to reflect, ask questions, and receive feedback from the leaders in an informal setting. This time also helps foster peer relationships and improves peer support, leading to greater knowledge gain. Commercial representatives should be invited to provide details on economic and technical aspects of products.

We initially thought sufficient time was allocated to crucial qPCR steps (i.e., standard development and pipetting proficiency). However, it became apparent that such activities should have received substantially more time. We strongly suggest that future qPCR training workshops include additional hands-on time to develop and enhance participant skills. This is especially critical for participants new to the field. However, participants were able to perform necessary data calculations to generate and interpret meaningful results. In the end, one week was sufficient time to expose and partially train even the most novice participants in the applied methods, but not sufficient to obtain the necessary proficiency required to fully implement the methodology in their own laboratories. Implementing rapid methods approved by USEPA to monitor recreational waters will require laboratory practice beyond the capabilities of a single workshop and will require the help and collaboration of all practitioners, regulators, and researcher/trainers.

We recognize that the organization of a single workshop cannot fully integrate rapid molecular methods into recreational water quality monitoring programs throughout the country. The workshop results showed that to truly implement the qPCR techniques into ongoing monitoring programs, additional training of personnel in their actual laboratories may be necessary. Key findings of this workshop demonstrated that scientists with no prior experience could be effectively trained in one week to conduct qPCR analysis. The success of this training was strengthened by collaborative interactions amongst newly trained practitioners, workshop leaders, and members of the water quality community.
